# Alpha-synuclein oligomers alter the spontaneous firing discharge of cultured midbrain neurons

**DOI:** 10.3389/fncel.2023.1078550

**Published:** 2023-01-20

**Authors:** Giulia Tomagra, Claudio Franchino, Federico Cesano, Giovanni Chiarion, Antonio de lure, Emilio Carbone, Paolo Calabresi, Luca Mesin, Barbara Picconi, Andrea Marcantoni, Valentina Carabelli

**Affiliations:** ^1^Drug Science Department, University of Torino, Turin, Italy; ^2^Nanostructured Interfaces and Surfaces Inter-Departmental Research Centre, Turin, Italy; ^3^Department of Chemistry and INSTM-UdR Torino, Turin, Italy; ^4^Mathematical Biology and Physiology, Department of Electronics and Telecommunications, Turin, Italy; ^5^Laboratory Experimental Neurophysiology, IRCCS San Raffaele Rome, Rome, Italy; ^6^Neurological Clinic, Fondazione Policlinico Universitario Agostino Gemelli IRCCS, Rome, Italy; ^7^Neurology, Department of Neuroscience, Faculty of Medicine, Università Cattolica del “Sacro Cuore,” Rome, Italy; ^8^Dipartimento di Scienze Umane e Promozione della Qualitá della Vita, Telematic University San Raffaele Roma, Rome, Italy

**Keywords:** alpha-synuclein, multi-electrodes arrays (MEA), midbrain dopamine neuron, Maximum of the Absolute Value of the Cross-Correlation (MAVCC), spontaneous firing activity

## Abstract

The aim of this work was to monitor the effects of extracellular α-synuclein on the firing activity of midbrain neurons dissociated from substantia nigra TH-GFP mice embryos and cultured on microelectrode arrays (MEA). We monitored the spontaneous firing discharge of the network for 21 days after plating and the role of glutamatergic and GABAergic inputs in regulating burst generation and network synchronism. Addition of GABA_*A*_, AMPA and NMDA antagonists did not suppress the spontaneous activity but allowed to identify three types of neurons that exhibited different modalities of firing and response to applied L-DOPA: high-rate (HR) neurons, low-rate pacemaking (LR-p), and low-rate non-pacemaking (LR-np) neurons. Most HR neurons were insensitive to L-DOPA, while the majority of LR-p neurons responded with a decrease of the firing discharge; less defined was the response of LR-np neurons. The effect of exogenous α-synuclein (α-syn) on the firing discharge of midbrain neurons was then studied by varying the exposure time (0–48 h) and the α-syn concentration (0.3–70 μM), while the formation of α-syn oligomers was monitored by means of AFM. Independently of the applied concentration, acute exposure to α-syn monomers did not exert any effect on the spontaneous firing rate of HR, LR-p, and LR-np neurons. On the contrary, after 48 h exposure, the firing activity was drastically altered at late developmental stages (14 days *in vitro*, DIV, neurons): α-syn oligomers progressively reduced the spontaneous firing discharge (IC_50_ = 1.03 μM), impaired burst generation and network synchronism, proportionally to the increased oligomer/monomer ratio. Different effects were found on early-stage developed neurons (9 DIV), whose firing discharge remained unaltered, regardless of the applied α-syn concentration and the exposure time. Our findings unravel, for the first time, the variable effects of exogenous α-syn at different stages of midbrain network development and provide new evidence for the early detection of neuronal function impairment associated to aggregated forms of α-syn.

## Introduction

Midbrain dopaminergic neurons play a role in several brain functions, including motor control, reward, motivation, learning, and cognition (Nieoullon, 2002; Wise, 2004; Iversen and Iversen, 2007), while dysfunction of the dopaminergic system is associated with brain disorders, such as Parkinson’s disease (PD), depression, schizophrenia, and addiction (Koob et al., 1998; Lewis and Lieberman, 2000; Dauer and Przedborski, 2003). The extreme vulnerability of *Substantia Nigra pars compacta* (*SNpc*) dopaminergic neurons, leading to neurodegeneration, may originate from a variety of different causes, including the repetitive onset of pacemaker-related calcium (Ca^2+^) transients, low Ca^2+^ buffering capability, apoptosis, altered gene expression, extended dendritic tree, high metabolic activity and α-synuclein (α-syn) misfolding. Several lines of evidence demonstrate that, before the onset of cell bodies’ neurodegenerative loss, synaptic accumulation of α-syn species may cause pre- and post-synaptic dysfunction (Houlden and Singleton, 2012; Froula et al., 2018; Ghiglieri et al., 2018; Durante et al., 2019; Tozzi et al., 2021). Though, the process of α-syn aggregation is not limited to the intracellular milieu (McNaught and Olanow, 2006), as demonstrated by the presence of α-syn oligomers in the extracellular medium of cultured neurons (Danzer et al., 2012; Emmanouilidou and Vekrellis, 2016) and in the cerebrospinal fluid (CSF) of PD patients (El-Agnaf et al., 2006; Eusebi et al., 2017). Thus, extracellular α-syn oligomeric aggregates are toxic species that precede fibril formation and may play a key role in progression of PD (Volpicelli-Daley et al., 2011; Tozzi et al., 2021).

The action of exogenous α-syn on the firing activity of cultured neurons depends on the exposure time, the applied concentration and the presence of monomers, oligomers or fibrils. For example, extracellular monomeric α-syn causes lipid rafts fragmentation that alters Ca^2+^ entry and neurotransmitter release (Emanuele et al., 2016). On cortical neurons, 48 h exposure to 5 μM recombinant α-syn causes the activation of Cav2.2 (N-type) calcium channels which move from raft to cholesterol-poor areas of the plasma membrane and increase neurotransmitter release (Ronzitti et al., 2014). At higher concentrations (50–100 μM), α-syn reduces the firing rate of the neuronal network by disrupting synaptic transmission despite preserving the neuronal ability to fire action potentials (Hassink et al., 2018). Intrastriatal injection of α-syn-preformed fibrils (a-syn-PFF) causes drastic increase in the spontaneous firing frequency at 12 weeks post-injection, while produces no effect at earlier stages (6 weeks) (Tozzi et al., 2021). All these findings underline the action of α-syn at a specific age of neuronal maturation, whereas our purpose was to monitor the time-dependent evolution of neuronal function impairment. We therefore used cultured midbrain neurons isolated from Substantia Nigra (SN) as a model to monitor the progressive impairment of the spontaneous firing activity following exposure to extracellular α-syn. Three different neuronal populations were identified on the basis of their firing discharge and responsiveness to applied L-DOPA: high-rate (HR) neurons, low-rate pacemaking (LR-p), and low-rate non-pacemaking (LR-np) neurons. By investigating the early alterations of midbrain neurons activity we could discover that α-syn oligomeric form drastically alter the firing pattern activity of neurons at late stages of development while preserving the excitability at earlier stages.

## Materials and methods

### Primary cultures of embryonic midbrain neurons

Mesencephalic dopamine neurons were obtained from SN, even though it is not possible to exclude the presence of dopamine neurons from VTA (Studer, 2001; Fath et al., 2009; Gaven et al., 2014). The ventral mesencephalon area was dissected from embryonic (E15) C57BL6 TH-GFP mice (Sawamoto et al., 2001; Matsushita et al., 2002). TH–GFP mice were kept heterozygous *via* breeding TH–GFP mice with C57BL/6 mice. All animals were housed under a 12-h light/dark cycle in an environmentally controlled room with food and water *ad libitum*. All experiments were conducted in accordance with the European Community’s Council Directive 2010/63/UE and approved by the Italian Ministry of Health and the Local Organism responsible for animal welfare at the University of Turin (Authorization 695/2020-PR).

The digestion buffer was composed of Hank’s balanced salt solution (HBSS, without CaCl_2_ and MgCl_2_), enriched with 0.18% glucose, 0.1% BSA, and 0.06% papain (Wortington, Lakewood, NJ, United States), 0.2% Dnase (SigmaAldrich) and it was stored at 4°C. Neurons were plated at final densities of 2,000 cells mm^–2^ on microelectrode arrays (MEAs). Cultured neurons were used at different DIV (days *in vitro*), depending on the experiment. MEAs were coated with poly-L-Lysine (0.1 mg/ml) and laminin (5 μg/ml) as substrate adhesion. Cells were incubated at 37°C in a 5% CO_2_ atmosphere, with Neuro Basal Medium containing 1% pen-strep, 1% ultra-glutamine, 2% B-27 plus, and 2.5% FBSd; pH 7.4.

To control glia proliferation, 5 μM 5-fluoro-2-deoxyuridine (FdU) from Sigma (SIGMA, St. Louis, MO, USA) was added into MEAs at DIV 4. To preserve dopaminergic neurons, 20 ng/ml recombinant human brain derived Neurotrophic Factor (BDNF, SIGMA) was added into MEAs at DIV 4.

### Micro-electrode arrays recordings

Micro-electrode arrays were purchased from Multichannel Systems (MCS, Reutlingen, Germany). MEAs consist of 60 TiN (titanium nitride) planar round electrodes (30 μm diameter; 200 μm center-to-center inter-electrode distance). MEA amplifier was kept inside an incubator with a controlled temperature (37°C) and humified atmosphere (i.e., gas flow of 5% CO_2_ and 95% O_2_). All measurements were performed by keeping the neurons in their culture medium. Acquired signals, after 1,200× amplification, were sampled at 10 kHz and acquired through the data acquisition hardware and MC-Rack software (MCS). For each trial, data acquisition was performed over 2 min recordings.

All experiments using drugs have been performed by adding the drugs to the culture medium under static conditions, without superfusion. For acute application, measurement started 5 min after drugs administration, in order to restore temperature and CO_2_ conditions inside the incubator.

L-DOPA was purchased from Sigma-Aldrich (SIGMA, St. Louis, MO, USA) and used at 20 μM final concentration. D_1_ (SCH-23390) and D_2_ (sulpiride) receptors antagonist were purchased from Sigma-Aldrich (SIGMA, St. Louis, MO, USA) and used at 10 μM final concentration.

The experiments with α-synuclein (S7820, Sigma-Aldrich, Merck Darmstadt Germany) were carried out using different concentrations (0.3, 0.5, 1, 3, and 70 μM). Recordings were performed in three conditions: acute applications (see below for details), 24 and 48 h after α-synuclein addition.

### Data acquisition and analysis

Data were exported using the MC-Rack software (MCS), after establishing a threshold that allows to discriminate the noise from the signal. Burst analysis and cross-correlation histograms (CCH) were performed using NeuroExplorer software (Nex technology, Colorado Springs, CO, USA). Burst activity was detected using the Poisson-surprise method (Cotterill and Eglen, 2019).

Spike synchronization was evaluated using two different methods. In the first approach, electrode synchronization was estimated by means of CCH. CCHs were realized by means of Neuroexplorer software (±500 ms, bin size 5 ms). Pairs of electrodes were considered synchronous if the CCH peak could be identified exceeding the 99% confidence limit (Abeles, 1982).

The second method, described in Chiarion and Mesin (2021b), identifies the spiking activities of different neurons by assuming that each neuron fires with a specific waveform. This algorithm has been applied on a representative MEA and aims to identify, for each electrode, the activity of different neurons, by breaking down the signal detected by each electrode as the sum of different contributions. This has been pursued through the following steps:

• only channels with a mean firing rate above 0.2 Hz were considered “active” and further processed (for the representative MEA that has been selected for this analysis, 37 channels out of 60 were found to be active);
• signals were band pass filtered in the range 300–3,000 Hz (Rey et al., 2015); 5th order low-pass and high-pass Chebyshev filters of type I have been used, with forward and reverse application to get zero-phase;
• data overcoming a threshold, equal to 5 times the mean absolute deviation (MAD), were identified and short epochs (2.5 ms duration) were collected.

The spike sorting algorithm identified, for each of the 37 active channels, waveforms reflecting the activity of 2 neurons (37 × 2 = 74 neurons in total), as described in Chiarion and Mesin (2021b).

A bin dimension of 5 ms was chosen for the analysis and the Maximum of the Absolute Value of the Cross-Correlation (MAVCC) was applied as an undirected index of functional connectivity (Chiarion and Mesin, 2021a), i.e., to consider both positive and negative correlations. In order to discard weaker connections, only MAVCC values above an absolute threshold of 0.1 were considered. Two topological indexes were evaluated: the node degree, defined as the number of the connections to a node (neuron) and the strength, which is the sum of the connections to a node. The strength represents the sum of the cross-correlations that each neuron has with respect to all other neurons (Bullmore and Sporns, 2009; Rubinov and Sporns, 2010).

### Statistics

Data are indicated in the text and figures as mean ± SE (standard error). Data distributions were assessed for normality using Shapiro–Wilk and Pearson normality test. All data are normal distributed.

Data were analyzed by one-way ANOVA followed by Bonferroni’s multiple comparison test using Origin Pro software. Overall statistical significance was set at *p* < 0.05, except where otherwise specified.

Coefficient of variation (CV) was calculated as the ratio of the standard deviation to the mean; it indicates the relative dispersion of data points in a data set around the mean.

### Atomic force microscopy (AFM) measurements

Atomic force microscopy measurements were carried out in the tapping mode by using a super sharp silicon cantilever (SSS-NCL, Nanosensors) having a length of 225 μm and a tip radius of curvature <5 nm. The cantilever was mounted on a Nanosurf Easyscan2 AFM instrument equipped with a high-resolution scan head. The instrument, shielded in an insulated enclosure, was placed on an antivibration platform. Before analysis, 2.5 μl of culture medium, incubated for 48 h with α-syn (final concentration 1 μM), was placed on a freshly cleaved mica support (V1 grade muscovite sheets) by using the drop casting method. The sample was investigated by AFM as soon as the atop mica surface became dry and within 2 h from the drop casting process. Several regions of the sample were AFM imaged to verify that the observed structures were representative of the whole sample. A control AFM acquisition was performed to verify the purity of the stock solution after dropping distilled water on the same freshly cleaved mica support. Scan speed was 0.3 Hz with an image resolution of 256 × 256 pixels. AFM images and data were processed and analyzed using the Gwyddion software (Neèas and Klapetek, 2012).

## Results

### Cultured midbrain neurons switch from sporadic firing to burst-driven activity during network maturation

**FIGURE 1:**
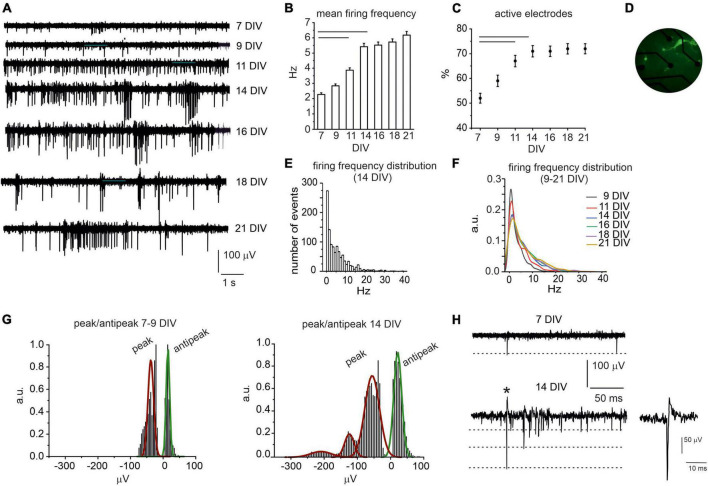
Spontaneous AP firing activity of midbrain neurons during network development. **(A)** Representative traces measured by the same electrode at different DIV (7–21). **(B)** Bar histogram of mean firing rate at different DIV. Statistical difference is indicated by the horizontal lines (7 DIV vs. 11 DIV, *p* < 0.0001; 7 DIV vs. 14 DIV, *p* < 0.0001). **(C)** Percentage increase of active electrodes versus time (7 DIV vs. 11 and 14 DIV *p* < 0.0001). **(D)** Photograph of midbrain TH-GFP neurons (14 DIV) cultured on microelectrode arrays (MEA). **(E)** Distribution of the spontaneous firing rate at 14 DIV. **(F)** Distribution of the spontaneous firing rate in the range 9–21 DIV (a.u. arbitrary units). **(G)** Distribution of negative peak and antipeak mean values for young (left) and elder neurons (right). **(H)** Representative AP traces at 7 DIV and 14 DIV; the dashed lines indicate the amplitude of the negative AP peaks. The single AP indicated by the asterisk is shown at higher magnification time scale in the inset. See (Marcantoni et al., 2022) for a detailed explanation of the AP time course.

The spontaneous activity of cultured midbrain neurons could be detected since 7 days *in vitro* (DIV) and was monitored until 21 DIV (Figure 1A). At the initial stages, action potential (AP) firing was mainly characterized by AP generated at low frequency: 7 and 9 DIV-neurons exhibited comparable mean firing rates, respectively, 2.3 ± 0.1 Hz and 2.8 ± 0.1 Hz (*N*_MEA_ = 15, *N*_ch_ = 590 and *N*_ch_ = 653, *p* = 0.09). Then, the AP firing rate significantly increased to 3.9 ± 0.2 Hz at 11 DIV (*N*_MEA_ = 15, N_ch_ = 718, *p* = 0.00712) and to 5.4 ± 0.2 Hz at 14 DIV (*N*_*MEA*_ = 15, N_ch_ = 766, *p* < 0.0001). From 16 to 21 DIV, no further statistical difference could be revealed respect to 14 DIV neurons: mean frequency at 21 DIV was 6.2 ± 0.2 Hz (*N*_MEA_ = 15, *N*_ch_ = 788, *p* = 0.09, Figure 1B). Correspondingly, the number of active electrodes (electrode measuring signals whose amplitude exceeded 2.5 times the background noise) progressively increased throughout culture maturation: active electrodes were 50% at 7 DIV and increased to 72% at 14 DIV (*p* = 0.008) to remain unaltered between 14 and 21 DIV (*N*_MEA_ = 15, Figure 1C). A high-magnification photograph of TH-GFP neurons at 14 DIV is shown in Figure 1D.

Thus, considering 14 DIV as a critical threshold for network maturation, we further investigated the firing rates distribution at this age. We found that the spontaneous firing frequency values were scattered from 0.1 to 40 Hz (Figure 1E). By setting a threshold at 4.5 Hz (Berretta et al., 2010), we found that 55% of neurons were spontaneously firing at low frequency (mean value 1.50 ± 0.04 Hz), whereas 45% fired at about a 6-fold higher frequency (10.0 ± 0.2 Hz). Notably, these data are in good agreement with those described in dopaminergic neurons of SN slices (Berretta et al., 2010) with a likely contamination of ventral tegmental neurons (Lacey et al., 1989). In Figure 1F, distributions of the firing frequency values in the range 9–21 DIV are superimposed. By comparing these distributions, it is worth noticing that firing rates exceeding 10 Hz progressively increase with age.

We next compared the waveform of the extracellular action potentials (Figures 1G, H) generated by early developed (<14 DIV) and elder neurons (>14 DIV). This analysis was limited to biphasic waves, since they represented the majority of events (>95% of cases). The remaining signals consisted in an early positive deflection and were no further investigated. Biphasic signals consisted in an early negative peak followed by a late positive deflection of smaller amplitude (defined as antipeak). We found that, for 7–9 DIV neurons, the negative peak had a mean amplitude of −40.9 ± 0.3 μV and was followed by an antipeak of 14.7 ± 0.1 μV. In elder neurons (14–21 DIV), the amplitude of the antipeak was still distributed around a unique mean value (21.5 ± 0.8 μV), while the distribution of negative peaks exhibited three mean values, centered at −55.0 ± 1.36 μV, −124.2 ± 4.1 μV and −209.6 ± 23.0 μV, respectively. A possible explanation is that during network maturation the ion conductance densities may vary considerably (Gold et al., 2006) and the burst-driven activity progressively increases, as discussed below.

**FIGURE 2:**
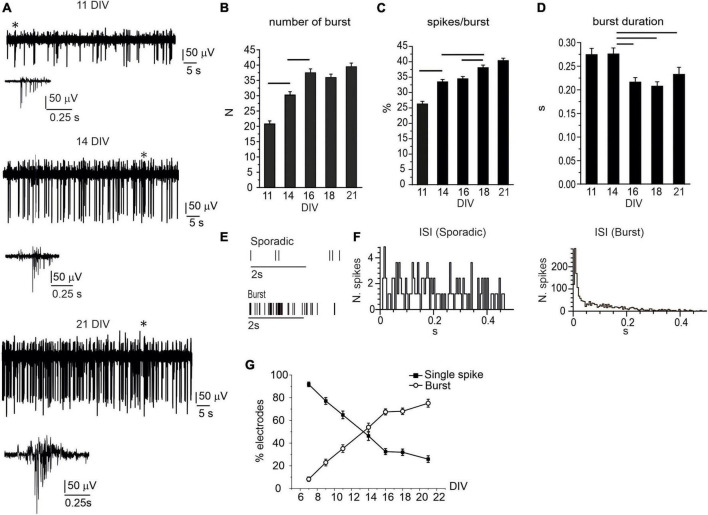
The burst activity progressively increases during network development. **(A)** Representative traces detected by the same electrode at 11, 14, and 21 DIV. Bursts (indicated by the asterisks) are shown at higher magnifications below the corresponding traces. **(B)** Bar plot of the mean number of bursts during 120 s recordings. Significant difference is indicated by the horizontal lines (11 DIV vs. 14 DIV, *p* < 0.0001; 14 DIV vs. 16 DIV, *p* < 0.0001). **(C)** Mean percentage of spikes within bursts, during 120 s recordings (11 DIV vs. 14 DIV, *p* < 0.0001; 14 DIV vs. 18 DIV, *p* < 0.0001, 16 DIV vs. 18 DIV, *p* = 0.01). **(D)** Mean burst duration in the range 11–21 DIV (14 DIV vs. 16 DIV, *p* = 0.0049; 14 DIV vs. 18 DIV, *p* < 0.0001; 14 DIV vs. 21 DIV, *p* = 0.05). **(E)** Raster plot for two representative electrodes detecting sporadic firing and burst activity. **(F)** Inter-spike interval (ISI) distribution shown for two representative microelectrode arrays (MEA) electrodes detecting either sporadic (left) or burst activity (right). **(G)** Percentage of electrodes detecting either sporadic or burst activity during network development.

*In vitro* network maturation was not limited to increased firing rates, relevant changes in the pattern activity (single-spike vs. burst-driven firing) occurred during the first 2 weeks in culture (Cohen et al., 2008; Gavello et al., 2012, 2018; Del Pino et al., 2020). In our experimental condition, single-spike firing was mainly detected at earlier stages of development (7–9 DIV), whereas, after 11 DIV, the occurrence of bursts progressively raised. For instance, in DA neurons, burst generation is associated with the activation of NMDARs and GABARs-mediated disinhibition (Tepper et al., 1995). The appearance of burst-driven activity, i.e., a group of APs occurring in rapid succession and followed by a period of quiescence (Zeldenrust et al., 2018), can be visualized in the traces shown in Figure 2A. Three exemplificative recordings, consecutively acquired from the same electrode, respectively, at 11, 14, and 21 DIV, are shown; whereas the progressive increase of burst generation is quantified in Figures 2B–D. Throughout network maturation, the number of bursts (monitored during 120 s recordings) progressively increased from 7 to 21 DIV, together with the percentage of spikes in burst during 120 s recordings, whereas burst duration significantly decreased from 0.27 ± 0.01 s to 0.20 ± 0.01 s (*p* = 0.004). Raster plots for two representative MEA channels detecting either sporadic spikes or burst activity are shown in Figure 2E.

To quantify the percentage of electrodes that were detecting either isolated AP or burst activity, we evaluated the interspike interval (ISI), calculated over 120 s recordings, for each electrode. Indeed, the ISI values allows to distinguish burst-driven activity from sporadic spikes firing (Chen et al., 2009; Rohrbacher et al., 2000). As shown in Figure 2F, for neurons that predominantly exhibit burst activity, the ISI distribution peaks at 0.15 ± 0.05 s, while for sporadic firing, the ISI distribution is rather uniform.

We followed this criterion (ISI distribution) for measuring the percentage of MEA electrodes that detected either bursts or sporadic spikes (*N*_MEA_ = 15). The percentage of electrodes exhibiting burst activity increased from 8.2 ± 1.9% (at 7 DIV) to 75.2 ± 3.5% (at 21 DIV, Figure 2G). On the contrary, the percentage of electrodes detecting isolated spikes decreased from 91.8 ± 2.0 to 25.9 ± 2.9. These two opposite trends cross each other at 14 DIV: after 14 DIV, the electrodes exhibiting burst firing prevail over those detecting single-spikes.

**FIGURE 3:**
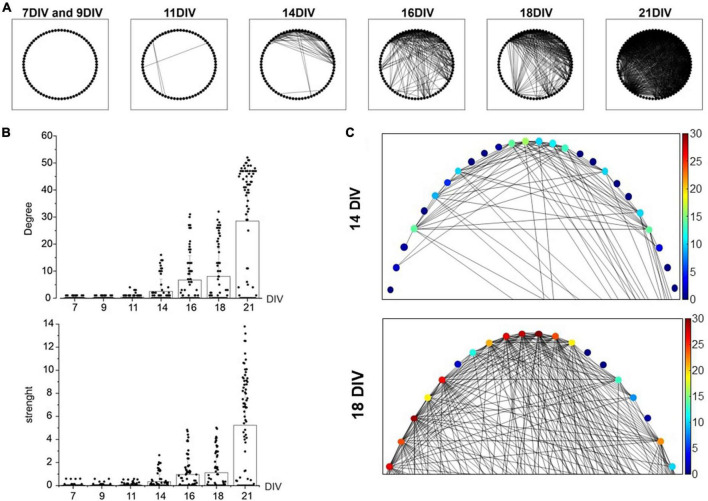
Cross-correlation increase evaluated through the Maximum of the Absolute Value of the Cross-Correlation (MAVCC). **(A)** The graphs for the adjacency matrixes of the culture for different days *in vitro* (DIV) is shown: each node (indicated as a black circle) represents a neuron and each edge (segment) represents the cross-correlation between the two neurons (MAVCC > 0.1). **(B)** Bar plots show the node degree and strength of each neuron. **(C)** An enlargement of the adjacency matrix reported in panel **(A)**. The algorithm has been applied to a representative microelectrode arrays (MEA), in which 37 active channels have been identified. Each channel detected the activity of 2 neurons (thus, *N* = 74 neurons were recognized). In order to explore possible functional connections between neurons, all possible couples were cross-correlated. Statistical differences (*p*-value <0.01) are found with a One-Way ANOVA (Tukey-HSD) test between different DIVs, both for node degree and strength indices.

After assessing the firing activity changes of the developing network (7–21 DIV), we monitored in the same temporal range the cross-correlation among MEA channels, as an index of synchronization among neurons. To this purpose, the “spike-sorting” algorithm was applied (see “Materials and methods”). In Figure 3A, a qualitative representation of the increased cross-correlated neurons is shown. Each neuron is represented as a point and the cross-correlation between pairs of neurons as a segment. The drastic increase of connections over time (7–21 DIV) is easily visible from the graph. A more quantitative evaluation of the increased cross-correlation is reported in Figure 3B that represents the distribution of the node degree (number of connections for each neuron) and of the strength (sum of the connections). The mean of the node degrees raised nearly 300-fold: from 0.1 ± 0.3 at 7 DIV to 28.5 ± 20.7 at 21 DIV, suggesting that the number of correlated neurons increased impressively with DIV. At the same time, the connections between neurons became stronger with time, as suggested by the strength distribution (sum of the MAVCC per neuron, see “Materials and methods”): mean values increased from 0.3 ± 0.6 (14 DIV) to 5.2 ± 4.3 (21 DIV). The increases of the node degree and the strength indicate an increasing cross-correlation during network maturation. In Figure 3C an enlargement at 14 DIV and 18 DIV of graphs reported in A is shown. The color scale indicates the increment (from blue to red) of connections in each node between neurons.

### Modulation of the firing rate and synchronism by GABAergic and glutamatergic input

After measuring the spontaneous firing activity and the network synchronism under control conditions, we next investigated the role of GABAergic and glutamatergic tone. Consecutive recordings were performed following the addition of picrotoxin (100 μM) and then APV (50 μM) + DNQX (20 μM) to the culture medium, to sequentially block GABA_A_ and NMDA + AMPA/kainate receptors. Drugs were tested on 14 DIV neurons since, as mentioned before, this day represents the critical stage of network maturation.

**FIGURE 4:**
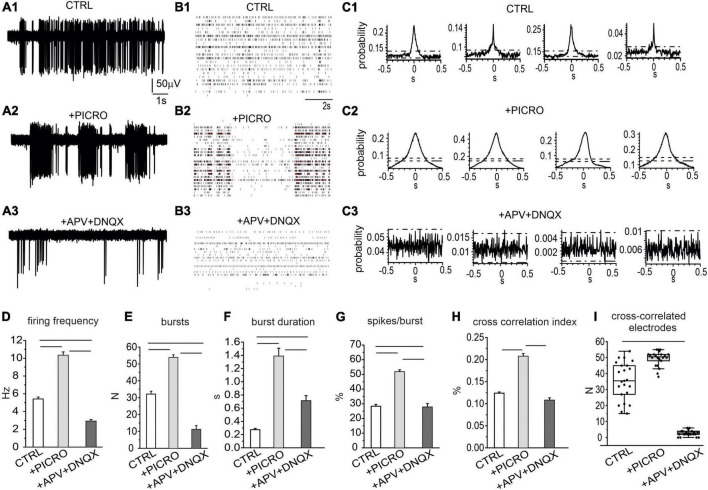
Role of excitatory and inhibitory neurons in regulating the spontaneous activity of the network. **(A1–A3)** Representative traces under control conditions, after addition of picrotoxin (100 μM) and of APV (50 μM) plus DNQX (20 μM), to selectively block GABA_A_, NMDA and AMPA/kainate receptors. **(B1–B3)** Raster plots in the three experimental conditions (control, + picrotoxin, + APV and DNQX). **(C1–C3)** Representative cross-correlograms (CCH) in control conditions, after the addition of picrotoxin and after the addition of APV and DNQX. **(D)** Mean firing rate in the three conditions (ctrl vs. picro *p* < 0.0001; picro vs. APV + DNQX *p* < 0.0001; ctrl vs. APV + DNQX, *p* < 0.0001). **(E)** Mean number of bursts during 120 s recordings (ctrl vs. picro, *p* < 0.0001; picro vs. APV + DNQX, *p* < 0.0001, ctrl vs. APV + DNQX, *p* < 0.0001). **(F)** Mean burst duration (ctrl vs. picro, *p* < 0.0001; picro vs. APV + DNQX, *p* < 0.0001; ctrl vs. APV + DNQX, *p* < 0.0001). **(G)** Percentage of bursts during 120 s recordings (ctrl vs. picro p < 0.0001, picro vs. APV + DNQX, p < 0.0001, ctrl vs. APV + DNQX, p = 0.0028). **(H)** Cross-correlation index (evaluated as the mean peak amplitude of the cross-correlogram, see C for the representative examples) (ctrl vs. picro *p* < 0.0001, picro vs. APV + DNQX, *p* < 0.0001). **(I)** Box-plot histogram showing the number of cross-correlated electrodes in control condition (CTRL) and after the sequential addition of receptor antagonists (picro, APV + DNQX), (ctrl vs. APV + DNQX, *p* < 0.0001).

Administration of picrotoxin to block GABA_A_ synapses (*N*_MEA_ = 8, *N*_ch_ = 773), caused almost a 100% increase of the firing rate: from 5.4 ± 0.2 Hz to 10.3 ± 0.4 Hz (Figures 4A, B). This effect was associated to an enhancement of the burst discharge, evaluated as increased number and burst duration, and percentage of spikes in the burst (Figures 4D–G). Subsequent administration of AP-V plus DNQX to block glutamatergic synapses drastically reduced the firing rate to 2.9 ± 0.2 Hz (*N*_MEA_ = 8, *N*_ch_ = 706, *p* < 0.00015), as well as the number of bursts, the burst duration and the percentage of spikes in bursts, confirming the key role of glutamate receptors in generating the bursting mode discharge (Johnson et al., 1992; Wang et al., 1994; Prisco et al., 2002). This reduction of the mean firing rate respect to control conditions suggests that a basal glutamatergic tone drives the network as already observed in primary cultured hippocampal neurons (Gavello et al., 2018). Differently from hippocampal neurons, in midbrain neurons NMDA and AMPA/kainate antagonists induced a reduction, but not a complete silencing of the spontaneous firing.

We next examined the temporal correlation between recorded trains of APs under control conditions or in the presence of GABA_A_, NMDA, and AMPA/kainate receptor antagonists. As a first overview of the altered firing mode when GABAergic and glutamatergic input are suppressed, raster plots are shown in Figures 4B1–B3. To provide a quantitative evaluation of network synchronization, CCH were used to plot the distribution of spike coincidence at a certain time shift (Jurjuţ et al., 2019). Representative CCHs for the three conditions are shown in Figures 4C1–C3. We found that under control conditions (without receptor antagonists), CCH peak was 0.12 ± 0.01 (Figure 4H), while addition of picrotoxin drastically potentiated the CCH peak up to 0.21 ± 0.01 (*p* < 0.0001), indicating an enhanced synchronization and suggesting that the GABAergic input exerted a negative control on network synchronization. Thus, activation of GABA_A_ receptors negatively affected both burst initiation and burst synchronization. The subsequent addition of APV and DNQX caused a drastic reduction of CCH peak (0.11 ± 0.01). As shown in Figure 4I, the number of synchronized electrodes was significantly increased by picrotoxin, but drastically reduced by the subsequent addition of APV together with DNQX. The mean number of synchronized electrodes was 35.5 ± 2.6 (control), 49.5 ± 1.0 (after the addition of picrotoxin) and then was reduced to 2.6 ± 0.4 after addition of APV + DNQX.

**FIGURE 5:**
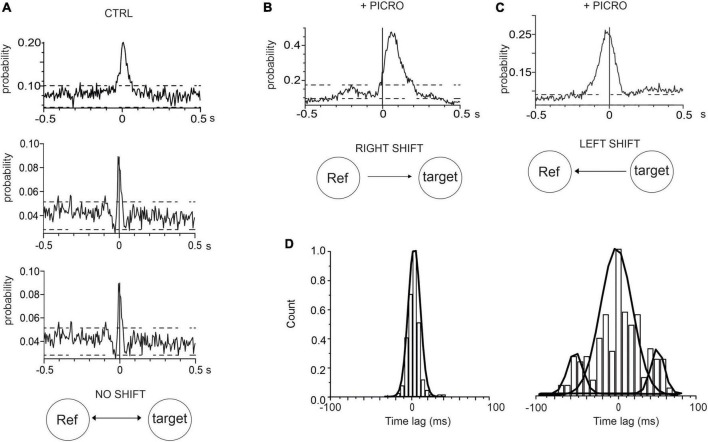
Cross-correlograms histograms (CCH) in control and in the presence of picrotoxin. **(A)** Three representative CCH under control conditions, whose peak is centered around 0 ms. **(B,C)** Representative CCH with picrotoxin: in this case the CCH peak is shifted from 0 ms (see text for details). **(D)** Time-lag distributions of CCH peak for controls (left) and with picrotoxin (right).

An additional information that can be drawn from the CCH is the identification of neurons whose activity leads the others. This information can be inferred by evaluating the position of the peak in the cross-correlogram histogram. In the case of synchronous firing, in which no neuron is leading the other, the peak in the CCH is centered at 0 ms offset, while the peak is shifted from 0 ms when the activation of one electrode is delayed (or anticipated) with respect to the other (Moore et al., 1970; Peyrache et al., 2012). By examining the position of the CCH peak, we found that in control conditions (for 14 DIV neurons, see representative examples in Figure 5A, the mean CCH peak was centered near 0 ms (0.4 ± 0.1 ms) This means that, independently of the random selection of reference and target electrodes, no delay offset exists between the spike trains recorded by the electrode pair (Maex et al., 2000). On the contrary, after adding picrotoxin, a shift of the CCH peak from 0 ms offset occurred, indicating that activation of one electrode is delayed (or anticipated) with respect to the other. This implies that neurons on the reference electrode may fire either faster or slower than the neurons on the target electrode. In this case, the CCH maximum peak probability exhibited a significant (positive or negative) time lag (Figures 5B, C). On average, the positive shift (64 ± 1 ms) indicates that the activation of the reference electrode precedes the activation of the target electrode, while the opposite occurs for the negative shift (−38 ± 2 ms). As shown in Figure 5D the time-lag peak distributions exhibit in control a unique peak, suggesting that the reference and target electrode detect the simultaneous activity of pairs of neurons. On the other hand, the presence of picrotoxin induces the generation of multiple peaks associated with the shift delay.

### Blocking the inhibitory and excitatory transmission uncovers the basal activity of pacemaking neurons

We next studied the firing properties of the spared network activity after blocking the inhibitory and excitatory transmission. Following the methods described in Berretta et al. (2010), we could distinguish different firing patterns of activity by measuring the spontaneous firing rate and the regularity of firing (Abeles, 1982; Bar-Gad et al., 2001).

**FIGURE 6:**
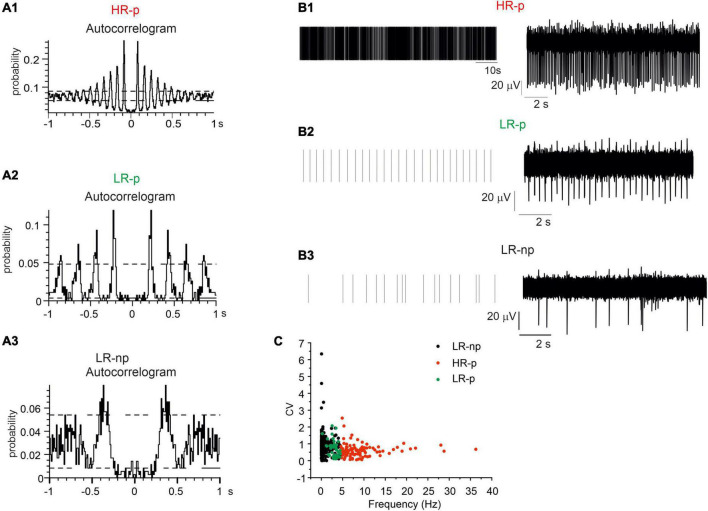
Identification of different midbrain neurons on the basis of their autocorrelograms. Representative autocorrelograms are shown, respectively, for: **(A1)** high-rate pacemaking neurons, **(A2)** low-rate pacemaking neurons, **(A3)** non-pacemaking neurons. **(B1–B3)** Raster plots and corresponding traces for a representative high-rate pacemaking neuron, low-rate pacemaking neurons and non-pacemaking neuron. **(C)** Scatter plot of firing rate vs. CV (coefficient of variation) evaluated by dividing the standard deviation of the inter-spike interval (ISI). The plot shows three different clusters of data corresponding to the three different populations low-rate non-pacemaking, High-Rate pacemaking, and low-rate pacemaking (LR-np, HR-p, and LR-p).

We found that 19% of neurons displayed high firing rates (mean value 9.2 ± 0.5 Hz; *N*_MEA_ = 6, *N*_ch_ = 82) and the corresponding autocorrelograms exhibited more than four consecutive peaks in a time window of 1 s. This feature represents an index of their highly regular activity (Figures 6A1–B1). These neurons were identified as HR-p (High-Rate pacemaking) (Berretta et al., 2010). The autocorrelation probability measured on the first of the repetitive peaks was 21.4 ± 0.7%. The coefficient of variation, CV, evaluated by dividing the standard deviation of the ISI by the mean ISI, was 0.90 ± 0.02 (Figure 6D). Conversely, another set of pacemaking neurons exhibited low frequency firing rates (2.9 ± 0.1 Hz) and at least three consecutive peaks in the autocorrelogram (Figures 6A2–B2): these were identified as Low-Rate pacemaking neurons (LR-p, *N*_MEA_ = 6, *N*_ch_ = 51). These subsets contributed to 12% of the spontaneously active neurons; the mean CV was 0.67 ± 0.07, while the autocorrelation probability measured on the first of the repetitive peaks was 18.2 ± 1.1%.

Finally, the remaining neurons, despite spontaneously active they exhibited LR-np activity. The mean spontaneous frequency was 0.76 ± 0.05 Hz and no consecutive peaks could be detected in the autocorrelogram (Figures 6A3–B3). The firing frequencies of HR-p, LR-P, and LR-np were significantly different (*p* < 0.001). Similarly to previous findings on SN slices (Berretta et al., 2010) also for cultured midbrain DA neurons, the scatter plots of CV versus the spontaneous firing frequency (Figure 6C) revealed the presence of three distinct clusters of data.

### Dual effects of L-DOPA on cultured midbrain DA neurons

**FIGURE 7:**
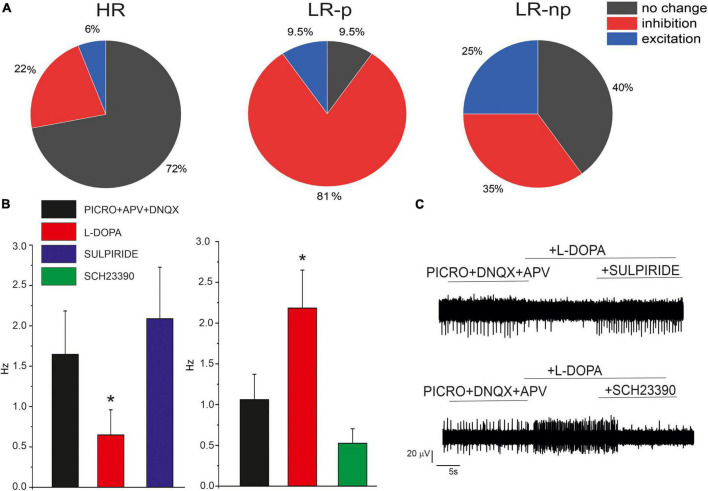
Effects of L-DOPA and D_1_/D_2_ antagonist on cultured midbrain DA neurons. **(A)** Pie plots of the percentage of neurons responding with inhibition, no change or excitation, in the HR, LR-p, and low-rate non-pacemaking (LR-np) subpopulations after L-DOPA administration. **(B)** Effect of D_1_ and D_2_ receptor antagonists, SCH-23390) and sulpiride after L-DOPA administration. **(C)** Representative AP traces in the indicated experimental conditions. *p < 005.

In order to investigate the modulation of the firing activity by released DA, we exogenously applied L-DOPA, which is internalized and then converted to DA by L-aromatic amino acid decarboxylase (Sebastianelli et al., 2008). In SN DA neurons, the synthesized DA is released from the somato-dendritic region and acts on D_2_ autoreceptors. Since D_2_-R are coupled to GIRK-2 channels, the modulatory autocrine loop causes membrane hyperpolarization and inhibition of AP firing activity (Mercuri et al., 1990). In addition to this inhibitory effect, a potentiating action of L-DOPA has been described in SN slices (Guatteo et al., 2013; Mannal et al., 2021) and in cultured midbrain neurons (Tomagra et al., 2019). Here we investigated the effects of L-DOPA in the presence of picrotoxin (100 μM), APV (50 μM) and DNQX (20 μM), to sequentially block GABA_A_ and NMDA + AMPA/kainate receptors. As shown in Figure 7, we found that L-DOPA exerted different effects on the different neuronal subpopulations of HR, LR-p, and LR-np neurons. In more details, most HR neurons (72%) did not respond to frequency changes after administration of L-DOPA. In agreement with similar findings on SN slices, we can argue that this subclass includes mainly GABAergic neurons, deriving either from SNc or SNr. In contrast, 81% of LR-p neurons responded with a decrease of the firing discharge, similarly to the typical response of SN DA neurons. Less defined was the response of LR-np neurons, as also reported in SN slices (Berretta et al., 2010).

In order to understand the different effects of L-DOPA, we used specific D_2_ and D_1_ antagonists (10 μM SCH-23390 and 10 μM sulpiride). We found that in 76% (*N*_MEA_ = 6) of neurons in which L-DOPA exerted an inhibitory effect, this was reversed by the D_2_ antagonist sulpiride, confirming that LR-pacemaking neurons are autocrinally inhibited by D_2_ receptors. Conversely, in 72% (*N*_MEA_ = 6) of cells which L-DOPA exerted a potentiating effect, this was reversed by the D_1_ antagonist SCH-23390, suggesting an involvement of D_1_ receptors, even though additional modulatory pathways cannot be excluded.

### Time- and dose-dependent depression of the firing rate by exogenous α-synuclein

The effects of extracellular α-syn were tested on late-stage developed neurons (14 DIV), by increasing the exposure time (from acute to chronic application up to 48 h) and by varying the α-syn concentration (from 0.3 to 70 μM) (Durante et al., 2019). Since we have shown that the basal firing rate of cultured midbrain neurons increased with time (Figure 1), the firing frequency of α-syn-treated neurons (48 h treatment) was always compared to control samples of the same DIV.

**FIGURE 8:**
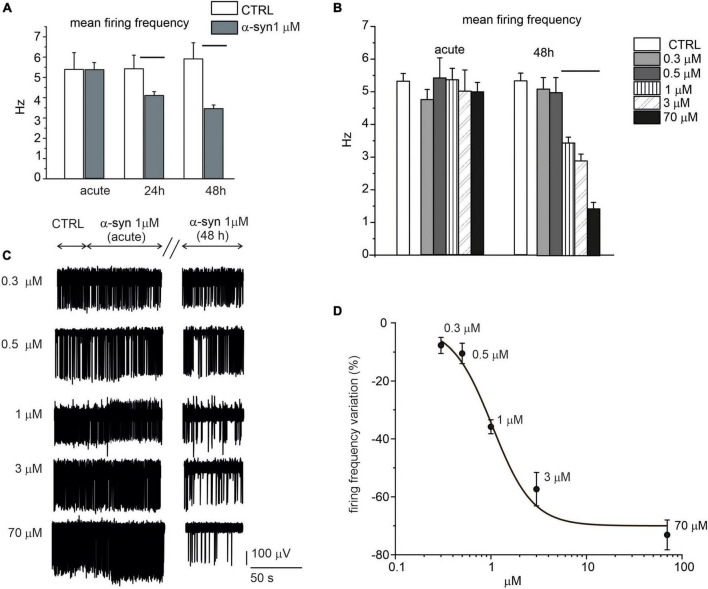
Time and dose-response effect of exogenous α-syn on the firing frequency of 14 DIV neurons. **(A)** Mean firing rate is shown in the bar histograms, respectively, without α-syn (control, CTRL), after acute application, after 24 h exposure (*p* = 0.004) and after 48 h exposure to α-syn 1 μM (*p* < 0.0001). **(B)** Mean values of the spontaneous firing frequency without α-syn (control, CTRL) and with increasing α-syn concentrations (ctrl vs. 1,3,70 μM *p* < 0.0001), either after acute application and after 48 h exposure. **(C)** Representative traces, detected by the same microelectrode arrays (MEA) electrode without α-syn (CTRL) and with increasing concentrations of α-syn. The effect of α-syn has been monitored immediately after α-syn application (acute), after 24 and 48 h, respectively. For each condition, recordings lasted 120 s; only a part of the recordings has been shown in panel C for better visualizing the events. **(D)** Dose response inhibition of the firing rate (Hill fit curve gave IC_50_ = 1.03 ± 0.25 μM).

When the network activity was recorded immediately after α-syn exposure (acute application), we could not reveal any difference in the firing frequency with respect to untreated control neurons, independently of the applied concentration (Figures 8A–C). On the contrary, firing rates were significantly altered by prolonged exposures to α-syn (24 and 48 h). Representative traces recorded by the same electrode show the spontaneous activity before (control), immediately after addition of α-syn (acute) and after 48 h exposure (Figure 8C). In more details, for 14 DIV neurons, the firing rate decreased from 5.3 ± 0.2 Hz (*N*_MEA_ = 16, *N*_ch_ = 615) to 4.1 ± 0.2 Hz (*N*_MEA_ = 16, *N*_ch_ = 621) after 24 h exposure (*p* = 0.004) and from 5.4 ± 0.3 Hz (*N*_MEA_ = 16, N_ch_ = 610) to 3.4 ± 0.2 Hz (*N*_MEA_ = 16, *N*_ch_ = 591) after 48 h exposure to α-syn (*p* < 0.0001). The dose-response curve was fitted with a Hill function and gave an IC_50_ = 1.0 ± 0.2 μM (Figure 8D). Thus, unless otherwise specified, we standardized the protocol by applying 1 μM α-syn for 48 h in the extracellular medium before recordings.

### Evaluation of the size distribution of α-synuclein oligomers

The morphology of α-syn oligomers was investigated by means of atomic force microscope (AFM) imaging. Data were acquired either in control conditions (CTRL), in which the culture medium was placed on a mica support for 48 h at 37°C, or in the presence of α-syn. In this case, α-syn (1 μM) was added to the culture medium and kept at 37°C for 48 h. A comparison of the 3D-AFM images in the two conditions (α-syn, CTRL) is shown, respectively, in Figures 8A, D.

**FIGURE 9:**
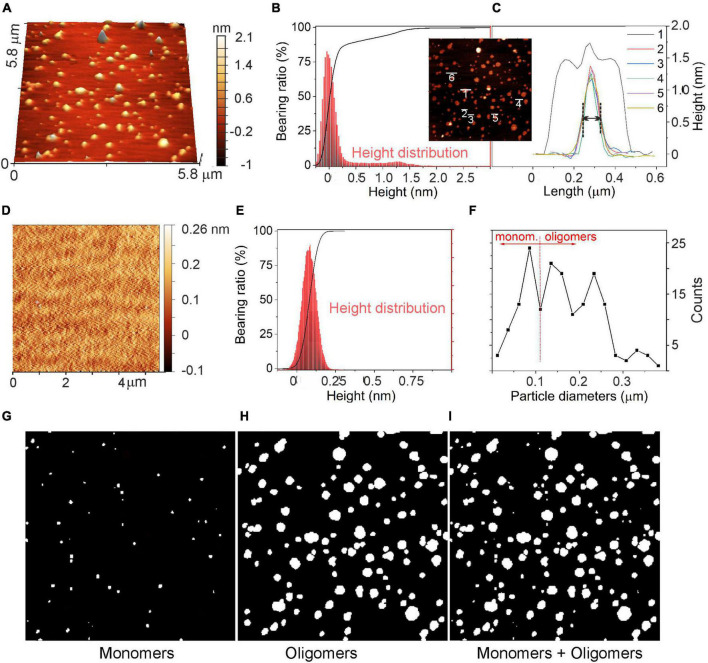
Evaluation of α-syn oligomeric species using atomic force microscopy (AFM). **(A)** AFM image of α-syn oligomeric species after incubation at 37°C for 48 h deposited on the mica support. **(B)** Abbott-Firestone curve (black line) and related height distribution (red histogram) in the presence of α-syn. **(C)** Height profiles of a few selected α-syn oligomers and their aggregates. In the inset: top-view of the AFM image shown in panel **(A)**. **(D)** AFM image of the mica support without α-syn and the related **(E)** Abbott-Firestone curve (black line) and height distribution (red histogram). **(F)** Diameter distribution of species imaged in panel **(A)**. **(G)** image of particles with diameters D ≤ 100 nm; **(H)** image of particles with diameters *D* > 100 nm (center image); and **(I)** all particles, respectively. *D* values refer to the diameters measured in the AFM images along the XY plane, as explained in the text.

The corresponding height distributions are shown in Figures 9B, C, E. In the presence of α-syn (Figure 9B), the histogram showed a first prevalent Gaussian profile with a maximum at about 0 nm ± 0.25 nm and a second broader profile at 1.25 ± 0.25 nm. It will be shown that these two heights are, respectively, indicative of the presence of the flat mica support and of the a-syn oligomeric species (Emadi et al., 2009; Bhak et al., 2018; van Diggelen et al., 2019). On the contrary, in control experiments (Figure 9E), a Gaussian-shaped height distribution centered at about 0.085 nm was obtained, corresponding to the roughness and noise associated with the flat mica support. A height variation of about 0.25 nm range was obtained. Such distribution exhibited a height distribution peaked at 50% of its height cumulative curve (Abbott-Firestone curve).

The height profiles of some selected particles are shown in the inset of Figures 9B, C. Isolated oligomers exhibited Gaussian-shaped profiles with heights of 1.2 nm and with full-with-at-half-maximum (FWHM) of approximately 100 nm in the lateral length (see profiles 2–6 in Figure 9C), while aggregated oligomers displayed a convoluted height profile, whose individual contributions were defined (profile 1, Figure 9C). Remarkably, as oligomeric species have sizes as small as the AFM tip radius (i.e., 5 nm), they exhibited ∼100 nm in length. As commonly observed for small objects, the length determined by AFM is of smaller accuracy and the size evaluation from the height profiles is more realistic (Lozano et al., 2019).

A statistical approach for the surface texture was then performed by computing the cumulative distribution function of the surface heights (Abbott-Firestone curve or bearing curve) (Borodich et al., 2020). As shown in Figure 9B (black curve), the cumulative curve of heights showed a peculiar shape with a first pseudo-vertical step in the ±0.25 nm interval for ∼90% of the bearing area, while a second increment corresponding to the remaining 10% of the surface indicates the presence of oligomers. This second increment is missing in the height distribution of CTRL experiment (Figure 9D). In addition, by comparing the surface roughness (Scarano et al., 2004) of the support, with or without α-syn (Figures 9A, D), scan area (5.8 μm × 5.8 μm), a mean roughness (*R*_a_) of 0,24 nm and an average root-mean-squared roughness (*R*_rms_) of 0,40 nm were calculated, while the freshly cleaved mica exhibited *R*_a_ and *R*_rms_ of ∼0.12 nm and ∼0.16 nm, respectively. Such cleaved mica roughness values refer to a flat, regular and clean surface, while the increase of *R*_a_ and of *R*_rms_ is further evidence for the presence of oligomers on the substrate surface.

Based on the AFM images shown in Figures 9 A–C, we can assume that monomers in the culture medium, after 48 h of incubation, evolve into the formation of oligomeric species, although some monomers may be still present. For this reason, a statistical approach (Figure 9F) was adopted to estimate the quantities of monomers and oligomers as obtained from the evaluation of diameters of particles imaged in Figure 9A. Such species have different lateral dimensions (i.e., diameters, *D*) that are *D* ≤ 100 nm (Figure 9G) for monomers and *D* > 100 nm (Figure 9H) for oligomeric species. From this result, we can conclude that the two species coexist, and that considering the entire size range, oligomer/monomer ratio can be estimated to be ∼1.8.

### Reduced network synchronization by α-synuclein

**FIGURE 10:**
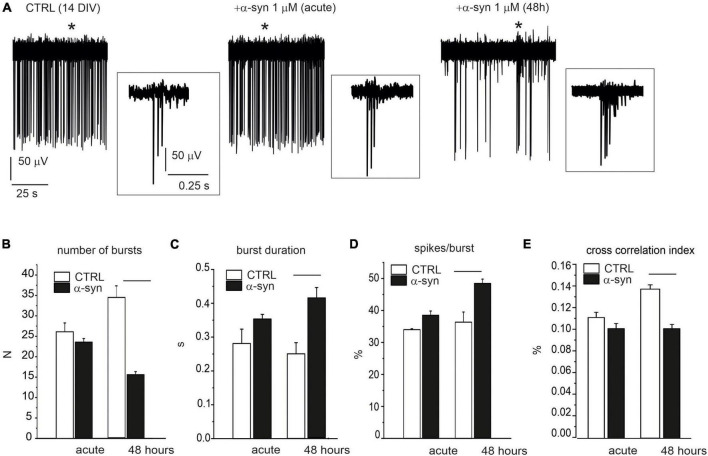
Impaired burst activity by α-syn (14 DIV neurons). **(A)** Single electrode recordings from one representative microelectrode arrays (MEA) under control conditions (without α-syn), immediately after α-syn addition (acute) and after 48 h exposure. Asterisks indicate the bursts that are shown in the insets at higher magnification scale. Part of the recording (120 s) has been shown. **(B–D)** Bar plots compare data without α-syn (CTRL), after acute addition of α-syn and after 48 h exposure. **(B)** Mean number of bursts (during 120 s recordings) (*p* < 0.0001). **(C)** Mean burst duration (*p* < 0.0001). **(D)** Mean percentage of spikes within bursts during 120 s recordings (*p* < 0.0001). **(E)** Mean cross-correlation histograms (CCH) peak amplitude (*p* < 0.0001).

We next investigated whether α-syn, besides reducing the spontaneous firing rate, also interfered with neuronal burst activity. The effects of α-syn (1 μM) were monitored on late-stage developed neurons both during acute treatment and after 48 h α-syn exposure (see Figure 10A for representative recordings). The impaired firing frequency was accompanied by a reduced number of bursts, from 24 ± 1 to 16 ± 1 (*p* < 0.0001, evaluated over 120 s recording, Figure 10B), an increased burst duration (from 0.32 ± 0.01 s to 0.41 ± 0.03 s, *p* < 0.0015, Figure 10C), and an increased percentage of spikes within the burst (from 37.5 ± 1.4% to 48.4 ± 1.4%, *p* < 0.0001, Figure 10D). We also observed that, after 48 h, the cross correlogram index decreased from 0.14 ± 0.01 to 0.10 ± 0.09, suggesting that α-syn not only slows down the basal firing frequency, but also significantly impairs burst activity and network synchronism.

### Exogenous α-synuclein preserves the firing mode of early-developed neurons

**FIGURE 11:**
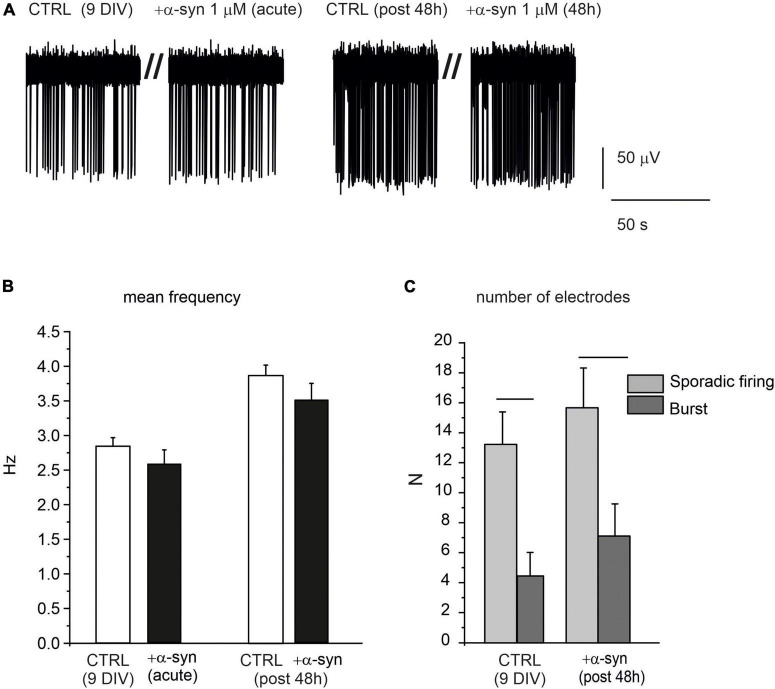
α-syn does not affect the spontaneous activity of early developed neurons (9 DIV). **(A)** Representative traces of spontaneously firing neurons at 9 DIV, under control conditions (without α-syn) and after 48 h exposure to α-syn. Recordings were performed over 120 s. Only a part of the recording is shown here to better visualize the events. **(B)** Bar histograms show the mean values of the firing frequency without α-syn (CTRL), after acute addition and after 48 h exposure to 1 μM α-syn. **(C)** Number of microelectrode arrays (MEA) electrodes detecting sporadic or burst activity. Statistical difference is indicated by the horizontal lines (CTRL, *p* = 0.005; α-syn post 48 h, *p* = 0.02).

Despite the dramatic effects exerted by α-syn on late-stage developed neurons, we found that when exogenous α-syn was applied on young neurons (9 DIV), it did not alter their spontaneous firing properties, either during acute application or after 48 h exposure. This is clearly shown in the representative traces reported in Figure 11A. The mean values of the spontaneous firing rate were 2.8 ± 0.1 s (without α-syn, control) and 2.6 ± 0.2 s (after acute exposure to α-syn, *p* = 0.9). After 48 h, control cells fired at 3.8 ± 0.1 s, while α-syn-treated cells fired at 3.1 ± 0.2 s (*p* > 0.04, Figure 11B). Then we focused on the pattern activity of 9 DIV neurons, to verify whether exposure to α-syn could alter the generation of sporadic firing, since, at this age, burst activity is still quite limited. To this purpose we monitored the number of MEA electrodes that were detecting either sporadic or burst activity, on the basis of the ISI distribution, as described in Figure 2. We found that, for neurons at early stage of development (9 DIV), the sporadic firing prevails over the generation of bursts, regardless of the presence of α-syn. As shown in Figure 11C, under control conditions, the number of MEA electrodes exhibiting sporadic firing were significantly higher then those exhibiting burst activity (sporadic: 13.22 ± 2.17, burst: 4.44 ± 1.57, *p* < 0.005, *N*_MEA_ = 9, *N*_ch_ = 718). Predominance of sporadic firing persisted after 48 h incubation with α-syn (sporadic: 15.7 ± 2.7, burst: 7.1 ± 2.1, *p* = 0.023), suggesting that the balance among sporadic and burst firing is not altered by α-syn at 9 DIV.

## Discussion

Since its discovery as the main constituent of Lewy bodies and neurites (Spillantini et al., 1997), many groups have focused on the detrimental effects of the intracellular aggregation of α-syn: impairment of midbrain neurons’ excitability (Hill et al., 2021), alteration of AP waveform (Yamamoto et al., 2019) and synaptic dysfunction following α-syn overexpression (Tozzi et al., 2016; Ghiglieri et al., 2018; Durante et al., 2019). Growing interest has been also directed to investigating the spreading of α-syn throughout the central nervous system (Braak et al., 2003), in order to identify its mechanism of action on different targets (El-Agnaf et al., 2006; Bridi and Hirth, 2018). For instance, intrastriatal injection of α-synuclein fibrils modifies the spontaneous firing of *SNpc* dopaminergic neurons, while preserving the activity of GABAergic neurons in *SNpr* (Tozzi et al., 2021). Similarly, dysregulated nigrostriatal function also impairs both forms of striatal synaptic plasticity (long-term potentiation and long-term depression). Since these alterations of neuronal function take place before neuronal death, they represent an interesting target for monitoring the early stages of neurodegeneration. Other effects in models overexpressing mutant α-syn include an impaired activity of type-A potassium channels, and, more recently, disruption of Ca^2+^ dynamics and reduced D_2_-autoreceptor inhibition (Subramaniam et al., 2014; Dagra et al., 2021; Lin et al., 2021).

Here we used, as a model system, midbrain neurons isolated from *SN* of TH-GFP embryos and cultured on MEAs for 21 DIV, with the specific aim of monitoring, in real time, the acute effects induced by exogenous α-syn aggregation on neuronal functionality, during neuronal development. Studying cultured neurons following isolation from SN is complicated by the presence of different neuronal populations, which derive either from the SN pars compacta and the SN pars reticulata, or from dopaminergic neurons of the VTA, as detailed in “Materials and methods.” Given the existing difficulties to specifically identify immunocytochemically the various subpopulations of SNpc, SNpr, and VTA neurons (Poulin et al., 2018) we opted for an electrophysiological characterization of neurons based on the analysis of firing frequency (HR, LR-p, and LR-np) and response to L-DOPA (see Figure 7).

Overall, our data demonstrate that the alterations of firing discharge is relevant after 48 h exposure to α-syn monomers, revealing reduced spontaneous AP firing, impaired network synchronization and burst generation in late-stage developed neurons. On the contrary, early-stage developed neurons do not exhibit any significant alteration of their functionality following exposure to α-syn.

### Spontaneous firing activity of cultured midbrain neurons

Our data on cultured midbrain neurons (Mannal et al., 2021; Tozzi et al., 2021) show clearly that basal firing rates progressively increase during network development and firing frequencies were normally distributed within a wide range of values. This variability is in good agreement with previous findings on *SNpc* slices (Berretta et al., 2010; Mannal et al., 2021) and can be also ascribed to the neurons dissociated from *substantia nigra pars reticulata* (*SNpr*) and VTA. Another relevant finding related to the network maturation concerns the modification of the AP shape extracellularly recorded by MEAs. The APs are characterized by a unique negative peak amplitude for early stage neurons and by two further distinct peaks of larger amplitude for elder neurons, which suggest either increased Nav channel densities during network maturation or the occurrence of burst activity that generates signals of variable amplitude.

The second observation concerns the switching from single-spike sporadic firing to burst-driven activity that occurs at 14 DIV and is associated to a more sustained network synchronization. The increased number of bursts along with culture maturation has been confirmed by examining the ISI distribution that allows to distinguish between the AP firing modes at the different stages of network development (Chen et al., 2009). Using the CCH and MAVCC approach, we could also find that network synchronization was particularly evident after suppressing the GABAergic component by the addition of picrotoxin. This is in good agreement with the observation that the transition from sporadic to burst firing is regulated by increased glutamatergic input, in which NMDA receptors may not be the only determinants (Chergui et al., 1993; Wang et al., 1994; Kitai et al., 1999). Indeed, metabotropic glutamate receptors (mGluRs) and AMPA may play a role, as well (Georges and Aston-Jones, 2002; Blythe et al., 2009; Dobi et al., 2010).

*In vivo* midbrain DA neurons are also constantly inhibited by active GABAergic tones (Tepper et al., 1995). This is different from what observed in hippocampal neurons, where inhibition of GABAergic synapses increases the network synchronization (Hofmann and Bading, 2006; Allio et al., 2015), suggesting that the contribution of GABAergic synapses on network excitability is strictly dependent on the ionic conductances regulating neuronal firing. In line with this, we also found that burst activity of the network is inhibited by GABA_A_R and potentiated by glutamatergic inputs. Further analysis, obtained by comparing the position of the CCH peak under control conditions and in the presence of picrotoxin, allows to determine the mean latency that two neurons set to excite and inhibit each other (Opalka et al., 2020). Through the MAVCC approach based on a spike-sorting algorithm, we could also confirm the progressive increase of synchronized neurons in the range 7–21 DIV.

Significantly, we could also show that following GABA_A_, NMDA and AMPA/KA receptors inhibition, the midbrain cultured neurons at 14 DIV exhibited firing modes that well reproduce those described in for *SN* DA neurons (Berretta et al., 2010). We could distinguish three different patterns of neuronal excitability: low- and high-rate pacemaking neurons and non-pacemaking neurons (see Figure 6 and Berretta et al., 2010). It should also be remarked that in our experimental midbrain conditions we could monitor the D_2_-mediated autoinhibition of AP firing, that represents the hallmark of *SN* DA neurons (Tomagra et al., 2019). In good agreement with data on *SN* slices, this inhibitory autocrine loop does not represents the unique effect exerted by released dopamine, since DA-induced potentiation of firing activity can also be revealed (Aversa et al., 2018; Mannal et al., 2021).

Overall, these findings suggest that cultured midbrain embryo neurons may be suitable for real time studies of neuronal activity alterations during α-syn aggregation (Volpicelli-Daley et al., 2014).

### Altered firing activity of midbrain neurons by α-synuclein oligomers

α-synuclein is a 140 aminoacid protein, enriched in the presynaptic terminal, that under particular conditions accumulates in aggregated forms, such as oligomers, protofibrils and fibrils within the neuronal inclusions typical of PD (Spillantini et al., 1997; Garcia-Reitboeck et al., 2013; Durante et al., 2019). Despite several evidence demonstrate fibrils toxicity, it remains still to be elucidated to which extent the conversion from monomeric to pathologic exogenous aggregates may alter neuronal function and play a role in the early phases of PD onset (Volpicelli-Daley et al., 2014).

To this purpose, we took advantage of cultured midbrain embryo neurons, dissociated from *SN* and interfaced with MEAs, to provide new insights on the potential detrimental role of exogenous α-syn on network functionality during development. Here we provide new evidence that α-syn oligomers do not impair the spontaneous firing frequency of early developed midbrain neurons, neither interferes with the spontaneous pattern activity of the network, even after 48 h exposure, likely due to low degree of neuronal contacts (Del Pino et al., 2020). We clearly demonstrate that 48 h incubation with α-syn (μM range) drastically altered network excitability of late-stage developed neurons (14–21 DIV), by impairing their burst activity and significantly reducing their spontaneous firing rate with significant attenuation of the synaptic transmission efficacy. This is in good agreement with data obtained from cortical networks, even if in that case experiments were performed using higher concentrations of α-syn; 24–48 h exposure to 50–100 μM α-syn reduced the network excitability as well as the network synchronism (Hassink et al., 2018). The molecular basis of the altered firing has still to be defined, although previous findings in cortical neurons show that α-syn, acting extracellularly, may increase Cav2.2 calcium channel function by altering the partitioning of surface-associated proteins and consequently modifying DA release (Ronzitti et al., 2014).

Inhibition of the spontaneous firing has been also observed on *SNpc* neurons, following exposure to 6-OHDA (Berretta et al., 2005; Qu et al., 2014; Wang et al., 2015), rotenone (Röper and Ashcroft, 1995) or MPTP (Carbone et al., 2017). Further evidence about the impaired DA neurons activity following α-syn exposure comes from recent data proving that α-syn pre-formed fibrils (PFF) reduce the percentage of spontaneously active neurons. This reduction is limited to *SNpc* at 6 weeks after α-syn-PFF injection, whereas the firing activity of GABAergic neurons of the *SNpr* is preserved (Tozzi et al., 2021). Alterations of the spontaneous firing discharge are associated with an initial neuronal loss and a deficit of striatal DA release, that could lead to consequent motor impairment. However, since under physiological conditions the bursting activity of DA neurons can sustain extracellular DA transients (Sulzer et al., 2016), it would be tempting to speculate that α-syn impaired capability of generating burst may affect DA release. Indeed, released DA exerts a negative-feedback on dopaminergic neurons excitability (Lacey et al., 1987; Tomagra et al., 2019). Further studies are needed to correlate the network synchronism to DA release at early stages of α-syn oligomers formation.

## Data availability statement

The raw data supporting the conclusions of this article will be made available by the authors, without undue reservation.

## Ethics statement

The animal study was reviewed and approved by the Local Organism responsible for animal welfare at the University of Turin (Authorization 695/2020-PR).

## Author contributions

GT: conception or design of the work, data acquisition and analysis, and approval and revision of the submitted version. CF: cell preparation and approval and revision of the submitted version. FC: conception or design of the work, data acquisition or analysis, and approval and revision of the submitted version. GC and LM: conception or design of the work, data analysis, and approval and revision of the submitted version. AdI: conception or design of the work and approval and revision of the submitted version. EC, PC, and BP: conception or design of the work, data analysis, and approval and critical revision of the submitted version. AM and VC: conception or design of the work, data acquisition and analysis, and approval and critical revision of the submitted version. All authors contributed to the article and approved the submitted version.
