# Corrigendum: Alpha-synuclein oligomers alter the spontaneous firing discharge of cultured midbrain neurons

**DOI:** 10.3389/fncel.2023.1176036

**Published:** 2023-03-21

**Authors:** Giulia Tomagra, Claudio Franchino, Federico Cesano, Giovanni Chiarion, Antonio de Iure, Emilio Carbone, Paolo Calabresi, Luca Mesin, Barbara Picconi, Andrea Marcantoni, Valentina Carabelli

**Affiliations:** ^1^Drug Science Department, University of Torino, Turin, Italy; ^2^Nanostructured Interfaces and Surfaces Inter-Departmental Research Centre, Turin, Italy; ^3^Department of Chemistry and INSTM-UdR Torino, Turin, Italy; ^4^Mathematical Biology and Physiology, Department of Electronics and Telecommunications, Turin, Italy; ^5^Laboratory Experimental Neurophysiology, IRCCS San Raffaele Rome, Rome, Italy; ^6^Neurological Clinic, Fondazione Policlinico Universitario Agostino Gemelli IRCCS, Rome, Italy; ^7^Neurology, Department of Neuroscience, Faculty of Medicine, Università Cattolica del “Sacro Cuore”, Rome, Italy; ^8^Dipartimento di Scienze Umane e Promozione della Qualitá della Vita, Telematic University San Raffaele Roma, Rome, Italy

**Keywords:** alpha-synuclein, multi-electrodes arrays (MEA), midbrain dopamine neuron, Maximum of the Absolute Value of the Cross-Correlation (MAVCC), spontaneous firing activity

In the published article, an author contribution was incorrectly written as [Ad]. The correct initial is [AdI].

In the published article, there was an error in the Funding statement. [This project was supported by Compagnia di San Paolo (Progetto Trapezio) and by Italian Miur]. The correct Funding statement appears below.

